# Combined Effect of Organic Acids and Modified Atmosphere Packaging on *Listeria monocytogenes* in Chicken Legs

**DOI:** 10.3390/ani10101818

**Published:** 2020-10-06

**Authors:** Elena Gonzalez-Fandos, Alba Martinez-Laorden, Iratxe Perez-Arnedo

**Affiliations:** Food Technology Department, University of La Rioja, CIVA Center. Madre de Dios Avenue 53, 26006 Logrono, Spain; alba.mar.lao@outlook.es (A.M.-L.); ipearn@gmail.com (I.P.-A.)

**Keywords:** food safety, *Listeria*, MAP, organic acids, foodborne pathogens, poultry

## Abstract

**Simple Summary:**

Chicken can be contaminated with pathogens such as *Listeria monocytogenes* and cause illness in consumers. The illness caused is called listeriosis, and it is a severe illness with high levels of mortality, particularly in susceptible individuals (pregnant women, neonates, the elderly and immunocompromised individuals). Consequently, it is important to control this pathogen in chicken meat. We evaluated the effect of packaging and natural compounds (citric, propionic and acetic acids) on this pathogen. We found that treatment of chicken with 2% propionic acid or 2% acetic acid can decrease *L. monocytogenes* counts in packaged chicken.

**Abstract:**

The combined effect of organic acid (citric, propionic or acetic acid) treatment and modified atmosphere packaging (MAP) on the growth of *L*. *monocytogenes* in chicken legs kept at 4 °C for 10 days was evaluated. Chicken legs were inoculated with *L. monocytogenes* and washed with either 2% citric, 2% propionic or 2% acetic acid solution or distilled water (control). Legs were packaged under the following conditions: air, vacuum, 80% N_2_/20% CO_2_, 60% N_2_/40% CO_2_ or 40% N_2_/60% CO_2_. The greatest *L. monocytogenes* growth reductions after treatment were observed in chicken legs washed with propionic acid (2.14 log units lower compared to control legs). The lowest growth rates of *L. monocytogenes* were found in samples washed with acetic acid and packaged in atmospheres containing CO_2_. An extended shelf life was observed in legs packaged in 40% N_2_/60% CO_2_, but these packaging conditions did not reduce *L. monocytogenes* growth. Consequently, it is necessary to design measures in order to control this bacterial pathogen. Washing of chicken with 2% propionic acid or 2% acetic acid can decrease *L. monocytogenes* counts in chicken packaged in MAP.

## 1. Introduction

Poultry is often identified as the source of foodborne outbreaks [1]. *Listeria monocytogenes* is frequently found in chicken and can grow on chicken meat. Moreover, the shelf life of fresh chicken is very limited, and it would be of interest to extend it [2].

Organic acids are used in foods because they are generally recognized as safe (GRAS) [3,4]. In order to reduce bacterial populations, high organic acid concentrations are needed, but these levels can adversely affect food quality [5]. Several investigations have addressed the application of organic acids on chicken [6,7]. 

The effectiveness of acetic, citric and propionic acids in reducing *L. monocytogenes* in chicken has been evaluated in early studies [8,9,10]. It was found that chicken legs washed with 2% acetic acid showed a decrease in *L. monocytogenes* counts and an extended shelf life of the samples by at least two days [10]. Previous studies demonstrated that washing with 2% citric acid or 2% propionic acid was effective against *L. monocytogenes* and reasonably preserved the sensorial quality [8,9].

The increased consumer demand for fresh products has encouraged the use of modified atmosphere packaging (MAP) as a method to prolong the shelf life of chicken [11,12]. However, there is a strong concern regarding the microbiological safety of these packaged foods because facultative anaerobic bacteria such as *L. monocytogenes* are able to grow under these conditions [12]. This bacterial species has the ability to grow at refrigeration temperatures. *L. monocytogenes* can grow on modified-atmosphere-packaged foods before spoilage can be detected, and thus measures have to be taken in order to control this bacterial pathogen in foods packaged under MAP.

The combined effect of MAP and organic acids on *L. monocytogenes* in chicken has been little studied, but some works have considered the effect of lactic or acetic acid and MAP [7,13]. Nevertheless, no works have been found on the combined effect of propionic or citric acid and MAP on *L. monocytogenes* in chicken meat.

This research was undertaken to evaluate the effectiveness of different organic acids (acetic, citric and propionic acid) to combat *L. monocytogenes* in packaged chicken legs stored at 4 °C.

## 2. Materials and Methods

### 2.1. Preparation of L. monocytogenes Inoculum and Inoculation of Chicken

The *L*. *monocytogenes* strain CECT 932 (equivalent to strain ATTCC 35152) was grown in Tryptone Soya Broth (Oxoid, Hampshire, UK) at 30 °C for 18 h to achieve a viable cell population of 9 log cfu/mL. The culture was then transferred to a sterile centrifuge bottle and centrifuged at 10,000× *g* for 10 min at 4 °C. The supernatant was decanted, and the pellet was resuspended in sterile 0.1% peptone solution (Merck, Darmstadt, Germany) (pH 6.2) by vortexing. The washing step was repeated twice. The suspension of washed cells was diluted in a sterile 0.1% peptone solution to obtain an appropriate cell concentration for inoculation.

Fresh chicken legs were obtained from a poultry processing plant (La Rioja, Spain). The legs were placed on crushed ice and transported to the laboratory. The legs were inoculated with *L*. *monocytogenes* by dipping them into a suspension of this pathogen (7 log cfu/mL) for 5 min at room temperature. After inoculation, the legs were removed, placed on a sterile surface and kept for 30 min at room temperature to allow the attachment of inoculated cells to the skin.

### 2.2. Experiment A—Packaging in Modified Atmosphere

After inoculation, each poultry leg was placed in a plastic bag (Dixie, Berm, Switzerland) and packaged under one of the following conditions: air (Batch A0), 80% N_2_/20% CO_2_ (Batch A20)_,_ 60% N_2_/40% CO_2_ (Batch A40), 40% N_2_/60% CO_2_ (Batch A60) and vacuum (Batch AV) (Praxair, Madrid, Spain). The film used had the following characteristics: O_2_ permeability less than 5 cm^3^/m^2^/24 h/atm, CO_2_ permeability less than 13 cm^3^/m^2^/24 h/atm at 25 °C and water vapor transmission rate less than 1.8 g/m^2^/24 h. The packaging equipment used was a Vaessen-Schoemake machine (Vaessen-Schoemake, Barcelona, Spain).

All the samples were kept at 4 °C for 10 days. Legs were evaluated on days 0 (after treatment), 1, 3, 6, 8 and 10. Each sampling day, six legs of each group were evaluated. Microbiological and sensorial analyses were conducted, and gas concentrations were determined.

### 2.3. Experiment B—Combined Treatments of Acetic, Citric or Propionic Acid and MAP

Samples were inoculated with *L. monocytogenes* as described in Section 2.1 and fractionated into four categories. Legs of each category were introduced for 5 min into different solutions: Group C into distilled water, Group AA into a 2% acetic acid solution, Group CA into a 2% citric acid solution and Group PA into a 2% propionic acid solution. Acetic, citric and propionic acid were obtained from Scharlau (Barcelona, Spain). After these treatments, the legs were removed and drained for 5 min. Legs from Batch C were packaged in air and stored at 4 °C.

Legs from groups AA, CA and PA were divided into five batches and packaged under the following conditions as described above: air, 80% N_2_/20% CO_2_, 60% N_2_/40% CO_2_, 40% N_2_/60% CO_2_ and vacuum. The washing and packaging conditions of each batch are shown in Table 1. Samples were evaluated on days 0, 1, 3, 6, 8, 10 and 15. Each sampling day, six legs of each group were used to carry out microbiological and sensorial analyses and to determine the gas concentrations inside the packages.

### 2.4. Sensorial Analysis

On the sampling days, six legs from each batch were evaluated for overall acceptability by a panel of nine members. A structured hedonic scale with numerical scores ranging from 7 (I like it very much) to 1 (I dislike it very much) was used. A score of 3 was considered the borderline of acceptability.

### 2.5. Microbiological Analyses and pH Determination

Each sample was prepared by aseptically removing 10 g of skin. The samples were blended in a sterile bag containing 90 mL of 0.1% sterile peptone water (Oxoid, Hampshire, England) and homogenized for 2 min (IUL, Barcelona, Spain). Serial dilutions in 0.1% sterile peptone were made. The psychrotrophs were determined on Plate Count Agar (Merck, Damstadt, Germany) with an incubation temperature of 7 °C for 10 days, using the pour plate method [14]. *Listeria* spp. were determined following the surface plate method on Palcam Agar at an incubation temperature of 30 °C for 48 h. Suspected colonies were identified according to the method described by Gonzalez-Fandos et al. [8]. Growth parameters (maximum growth rate and lag phase) were calculated by using the ComBase application and entering the data into the DMFit tool. The goodness of fit was evaluated using the coefficient of determination (R^2^).

A Crison model 2002 pH meter with a penetration electrode was used for measurements of pH (Crison, Barcelona, Spain).

### 2.6. Gas Determination

Determination of carbon dioxide and oxygen was carried out using an O_2_ and CO_2_ headspace gas analyzer (Check-mate model 9900, PBI-Dansensor, Ringsted, Denmark).

### 2.7. Statistical Analysis

Plate count data were transformed to logarithms before their statistical analysis. The experimental design was a factorial experiment (4 × 5) in which the factors were the washing treatment and the gas atmosphere composition with six repetitions. Analysis of variance was carried out using the SYSTAT program for Windows, Statistics version 5.0 (Evanston, IL, USA). Tukey’s test for comparison of means was performed using the same program. The level of significance was determined at *p* < 0.05.

## 3. Results

### 3.1. Modified Atmosphere Packaging

**Figure 1:**
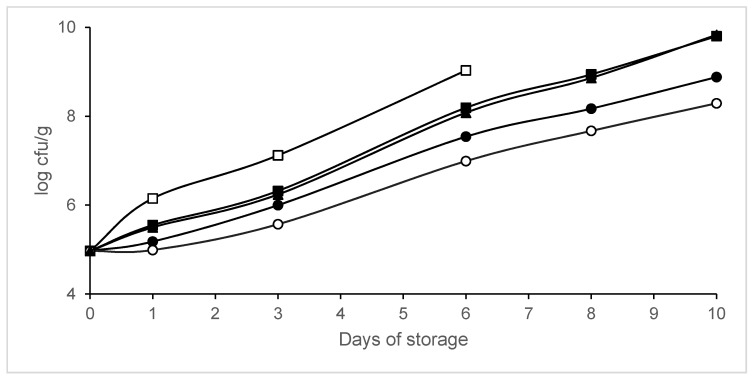
Changes in psychrotroph counts in packaged legs. Packaging conditions: Batch A0, air (□); Batch A20, 80% N_2_/20% CO_2_ (▪); Batch A40, 60% N_2_/40% CO_2_ (∙); Batch A60, 40% N_2_/60% CO_2_ (◦); Batch AV, vacuum (▲). Data shown are each the mean value of six determinations.

The effect of MAP on psychrotroph populations is shown in Figure 1. Significant differences (*p* < 0.05) in psychrotroph populations were found between the samples packaged in MAP and the samples packaged in air (control legs), except on day 0. Psychrotroph growth reductions of between 1.16 and 2.04 log units were observed in samples packaged in 40% N_2_/60% CO_2_ compared to the control samples. The growth parameters (maximum growth rate and lag phase) estimated by ComBase are summarized in Table 2. No lag phase for psychrotrophs was detected in legs packaged in air. The maximum growth rate decreased and the lag phase increased when the CO_2_ concentration increased. The legs packaged in vacuum showed a higher maximum growth rate and a shorter lag phase for psychrotroph growth compared to those packaged in 40% N_2_/60% CO_2_ or 60% N_2_/40% CO_2_. The lower maximum growth rate and longer lag phase were found for psychrotrophs in legs packaged in 40% N_2_/60% CO_2_. The growth parameters were adequately described, since R^2^ values obtained were above 0.95.

**Figure 2:**
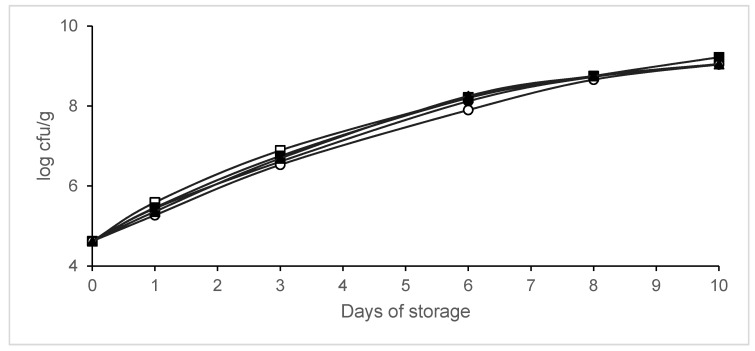
Effect of packaging conditions on the growth of *L. monocytogenes* on chicken legs packaged in modified atmospheres. Packaging conditions: Batch A0, air (□); Batch A20, 80% N_2_/20% CO_2_ (▪); Batch A40, 60% N_2_/40% CO_2_ (∙); Batch A60, 40% N_2_/60% CO_2_ (◦); Batch AV, vacuum (▲). Data shown are each the mean value of six determinations.

The effect of MAP on the growth of *L*. *monocytogenes* inoculated into legs is shown in Figure 2. No significant differences (*p* > 0.05) in these pathogen counts were found between samples packaged in MAP and control legs. No lag phase was detected in *L*. *monocytogenes* growth on samples packaged in different conditions. However, the maximum growth rate decreased when CO_2_ concentration increased (Table 2). The growth parameters were adequately described, as indicated by R^2^ values above 0.98.

**Figure 3:**
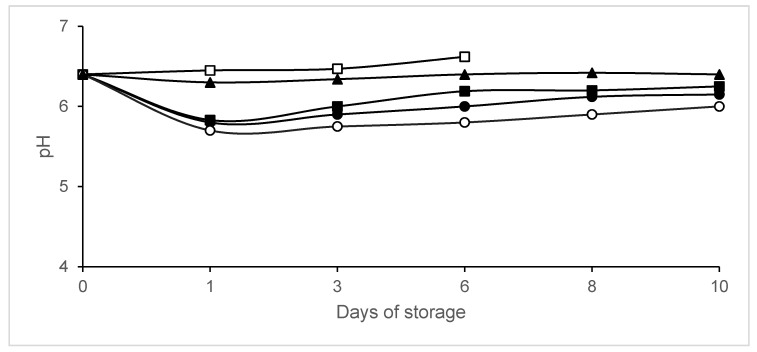
The evolution of pH in chicken legs packaged in modified atmospheres. Packaging conditions: Batch A0, air (□); Batch A20, 80% N_2_/20% CO_2_ (▪); Batch A40, 60% N_2_/40% CO_2_ (∙); Batch A60, 40% N_2_/60% CO_2_ (◦); Batch AV, vacuum (▲). Data shown are each the mean value of six determinations.

The pH value decreased when the CO_2_ concentration increased (Figure 3). Significant differences (*p* < 0.05) were observed in pH values between legs packaged in atmospheres containing CO_2_ and those packaged in vacuum or air, except on day 0. On day 1, pH in legs packaged in 40% N_2_/60% CO_2_ was 5.7 ± 0.01, while that in control legs was 6.45 ± 0.16.

Changes in package atmospheres (O_2_, CO_2_, N_2_) were not significant (*p* > 0.05) during storage among samples packaged in the same conditions. After 3 days of storage, CO_2_ and O_2_ levels remained steady.

**Figure 4:**
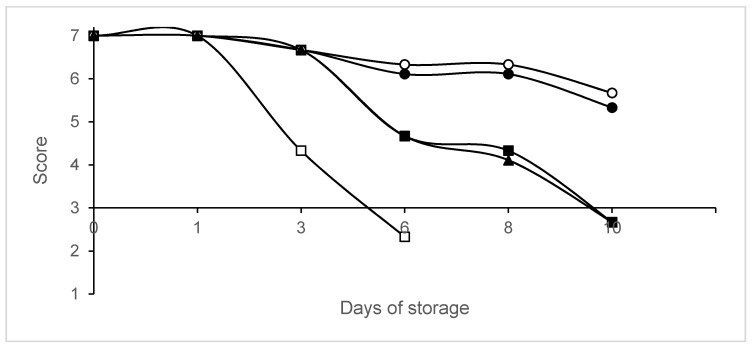
Changes in sensorial scores in packaged legs. Packaging conditions: Batch A0, air (□); Batch A20, 80% N_2_/20% CO_2_ (▪); Batch A40, 60% N_2_/40% CO_2_ (∙); Batch A60, 40% N_2_/60% CO_2_ (◦); Batch AV, vacuum (▲). Data shown are each the mean value of six determinations.

The sensorial acceptability of packaged legs is shown in Figure 4. Control legs were unacceptable on day 6, while those packaged in 80% N_2_/20% CO_2_ and vacuum were rejected on day 10. The samples packaged in 60% N_2_/40% CO_2_ and 40% N_2_/60% CO_2_ remained acceptable on day 10.

### 3.2. Combined Treatments of Organic Acids and MAP

**Figure 5:**
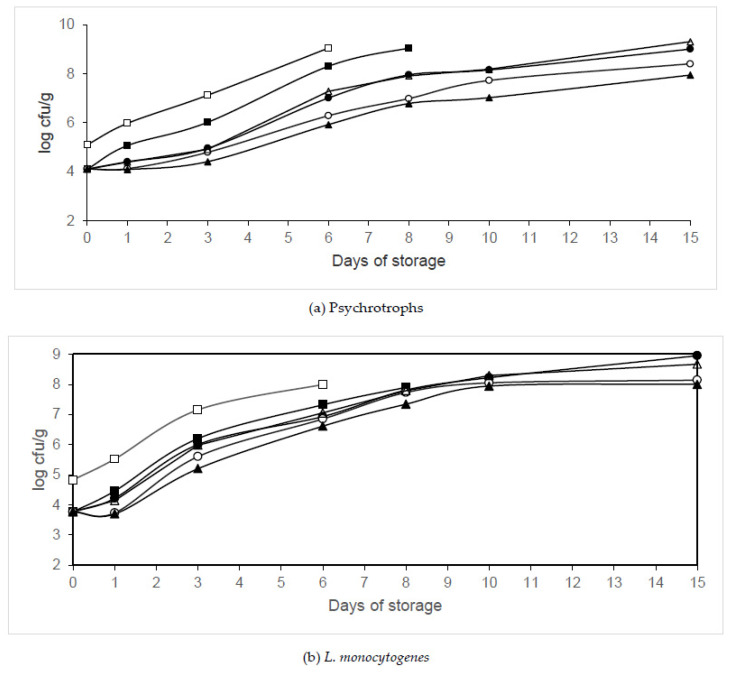
Changes in psychrotroph and *L. monocytogenes* counts in chicken legs treated with citric acid and packaged under modified atmosphere packaging (MAP). Treatment conditions: Batch C, distilled water and packaging in air (□); Batch CA, 2% citric acid and packaging in air (▪); Batch CAM20, 2% citric acid and packaging in 80% N_2_/20% CO_2_ (∙); Batch CAM40, 2% citric acid and packaging in 60% N_2_/40% CO_2_ (◦); Batch CAM60, 2% citric acid and packaging in 40% N_2_/60% CO_2_ (▲); Batch CAV (2% citric acid and packaging under vacuum (Δ). (**a**) Psychrotrophs. (**b**) *L. monocytogenes.* Data shown are each the mean value of six determinations.

**Figure 6:**
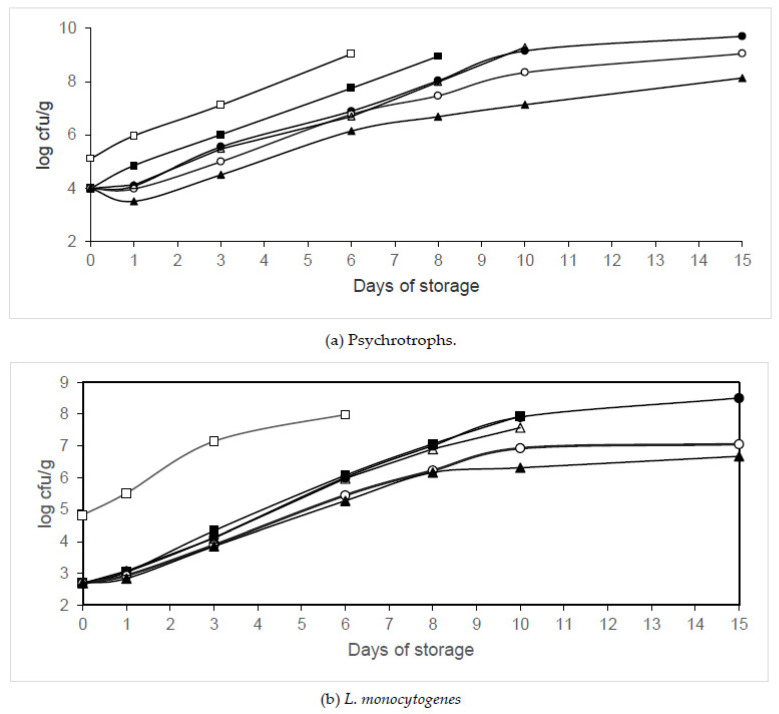
Changes in psychrotroph and *L. monocytogenes* counts in chicken legs treated with propionic acid and packaged under MAP. Treatment conditions: Batch C, distilled water and packaging in air (□); Batch PA, 2% propionic acid and packaging in air (▪); Batch PAM20, 2% propionic acid and packaging in 80% N_2_/20% CO_2_ (∙); Batch PAM40, 2% propionic acid and packaging in 60% N_2_/40% CO_2_ (◦); Batch PAM60, 2% propionic acid and packaging in 40% N_2_/60% CO_2_ (▲); Batch PAV, 2% propionic acid and packaging under vacuum (Δ). (**a**) Psychrotrophs. (**b**) *L. monocytogenes.* Data shown are each the mean value of six determinations.

**Figure 7:**
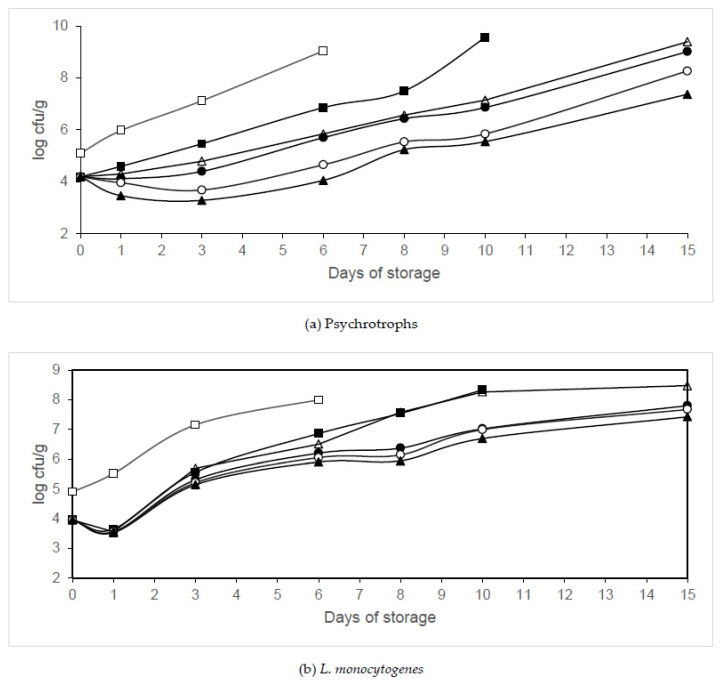
Changes in psychrotroph and *L. monocytogenes* counts in chicken legs treated with acetic acid and packaged under MAP. Treatment conditions: Batch C, distilled water and packaging in air (□); Batch AA, 2% acetic acid and packaging in air (▪); Batch AAM20, 2% acetic acid and packaging in 80% N_2_/20% CO_2_ (∙); Batch AAM40, 2% acetic acid and packaging in 60% N_2_/40% CO_2_ (◦); Batch AAM60, 2% acetic acid and packaging in 40% N_2_/60% CO_2_ (▲); Batch AAV, 2% acetic acid and packaging under vacuum (Δ). (**a**) Psychrotrophs. (**b**) *L. monocytogenes.* Data shown are each the mean value of six determinations.

The effects of different organic acids (citric, propionic and acetic acids) on psychrotroph populations in packaged legs are shown in Figure 5a, Figure 6a and Figure 7a. Significant growth reductions (*p* < 0.05) in psychrotrophs were observed between the samples treated with organic acids packaged in air or MAP and those not treated with organic acids. Significant growth reductions (*p* < 0.05) in psychrotrophs were also observed between the samples treated with organic acids packaged in MAP and those treated and packaged in air. The fastest increase in psychrotroph growth was observed in the air-packaged legs that were not treated with organic acids (Batch C). The samples packaged in 40% N_2_/60% CO_2_ and washed with 2% acetic acid (Batch AA60) showed the lowest psychrotroph counts. After treatment with citric, propionic, and acetic acids, psychrotroph growth reductions of 0.99, 1.11 and 0.91 log units, respectively, were observed. The treatment with 2% acetic acid and packaging in 40% N_2_/60% CO_2_ decreased psychrotroph growth by between 2.51 and 4.99 log units compared to the untreated samples. The growth parameters (maximum growth rate and lag phase) estimated by ComBase are summarized in Table 3. No lag phase was found in psychrotroph growth in samples washed with citric or propionic acid and packaged in air. However, the treatment with acetic acid and packaging in air extended the lag phase of psychrotrophs. The maximum growth rate decreased and the lag phase increased when the CO_2_ concentration increased. A significantly longer (*p* < 0.05) lag phase of psychrotrophs was found in legs washed with 2% acetic acid and packaged in 60% N_2_/40% CO_2_ or 40% N_2_/60% CO_2_ when compared to the other conditions tested. The lowest maximum growth rate of psychrotrophs was observed in legs washed with 2% propionic acid and packaged in 40% N_2_/60% CO_2_. The growth parameters were adequately described since R^2^ values were above 0.95. Washing treatment and packaging in modified atmospheres significantly affected psychrotroph growth (*p* < 0.05).

Figure 5b, Figure 6b and Figure 7b show the effects of different organic acids on the growth of *L*. *monocytogenes* in poultry samples packaged in different conditions. After treatment (day 0) with citric, propionic and acetic acids, *L. monocytogenes* populations were observed to decrease by 1.06, 2.14 and 0.88 log units, respectively, compared to the control samples. Significant reductions (*p* < 0.05) in *L. monocytogenes* growth were found in the samples washed with citric acid and packaged in 60% N_2_/40% CO_2_ or 40% N_2_/60% CO_2_ when compared to those washed with this organic acid and packaged in air, after 1 day of storage. Significant reductions (*p* < 0.05) in *L. monocytogenes* growth were found in the samples washed with propionic acid and packaged in 60% N_2_/40% CO_2_ or 40% N_2_/60% CO_2_ when compared to those treated with this organic acid and packaged in air, after 3 days of storage. Significant growth reductions (*p* < 0.05) in *L. monocytogenes* were found in the samples washed with acetic acid and packaged in 60% N_2_/40% CO_2_ or 40% N_2_/60% CO_2_ when compared to those washed with this organic acid and packaged in air, after 3 days of storage. Until day 6, the lowest *L. monocytogenes* populations were found in legs washed with 2% propionic acid and packaged in 40% N_2_/60% CO_2_. The treatment with 2% propionic acid and packaging in 40% N_2_/60% CO_2_ reduced *L. monocytogenes* growth by between 2.14 and 3.3 log units compared to the control samples. The treatment with 2% acetic acid and packaging in 40% N_2_/60% CO_2_ reduced *L. monocytogenes* growth by between 0.88 and 2.09 log units compared to the control samples throughout storage. The growth parameters (maximum growth rate and lag phase) estimated by ComBase are summarized in Table 3. No lag phase was found in *L. monocytogenes* growth in legs treated with citric or acetic acid and packaged in air. However, the treatment with propionic acid and packaging in air extended the lag phase of this pathogen. The maximum growth rate decreased and the lag phase increased when the CO_2_ concentration increased. A significantly lower (*p* < 0.05) growth rate of *L. monocytogenes* was found in legs treated with 2% acetic acid and packaged in modified atmospheres containing CO_2_ than in those treated with citric or acetic acid and packaged under MAP. The R^2^ values obtained were above 0.89. Washing treatment and packaging in modified atmospheres significantly affected *L. monocytogenes* growth (*p* < 0.05).

**Figure 8:**
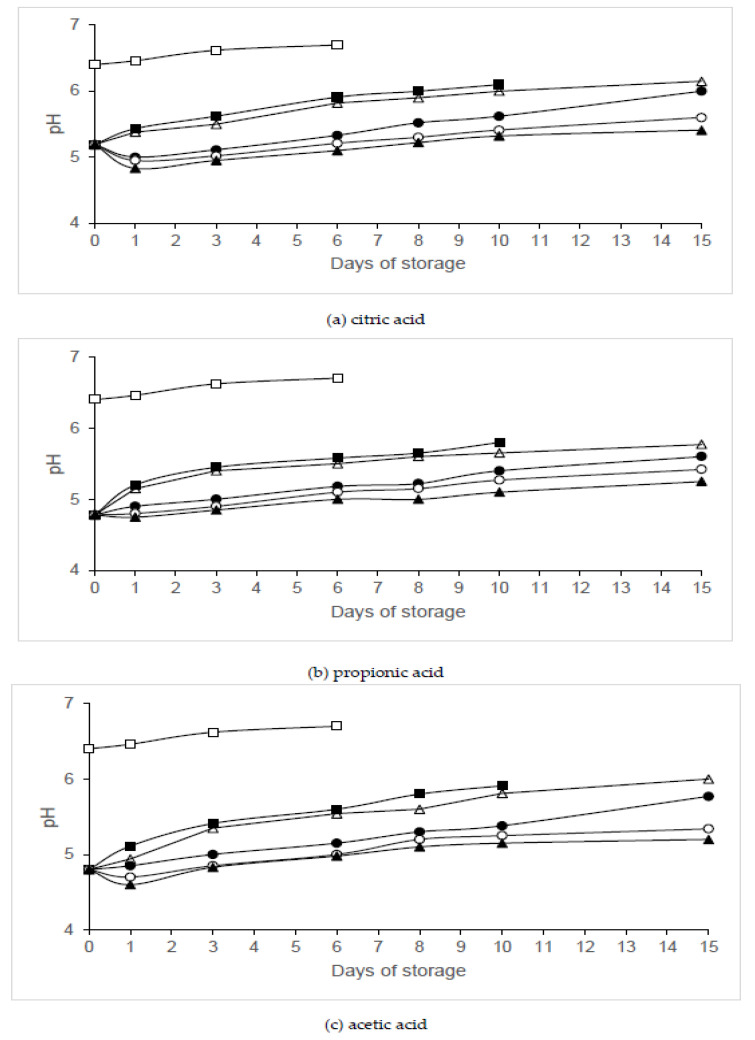
Evolution of pH in chicken legs treated with organic acids and packaged under modified atmospheres. Treatment conditions: Batch C, distilled water and packaging in air (□); 2% organic acid, packaging in air (▪); 2% organic acid, packaging in 80% N_2_/20% CO_2_ (∙); 2% organic acid, packaging in 60% N_2_/40% CO_2_ (◦); 2% organic acid, packaging in 40% N_2_/60% CO_2_ (▲); 2% organic acid, packaging under vacuum (Δ). (**a**) Citric acid; (**b**) propionic acid; (**c**) acetic acid. Data shown are each the mean value of six determinations.

Figure 8 shows the pH values of chicken legs treated with organic acid and packaged under MAP. Initial pH value in control samples was 6.4 ± 0.11, while pH values in legs washed with solutions containing 2% citric, propionic or acetic acid (day 0) were 5.19 ± 0.09, 4.78 ± 0.06 and 4.80 ± 0.10, respectively. Significant reductions (*p* < 0.05) in pH value were observed in samples washed with an organic acid and packaged in 60% N_2_/40% CO_2_ or 40% N_2_/60% CO_2_ when compared to those washed with the same organic acid and packaged in air or vacuum, except on day 0. CO_2_ concentrations decreased by about 4% during storage in all packaged samples. After 3 days of storage, CO_2_ concentrations remained unchanged

**Figure 9:**
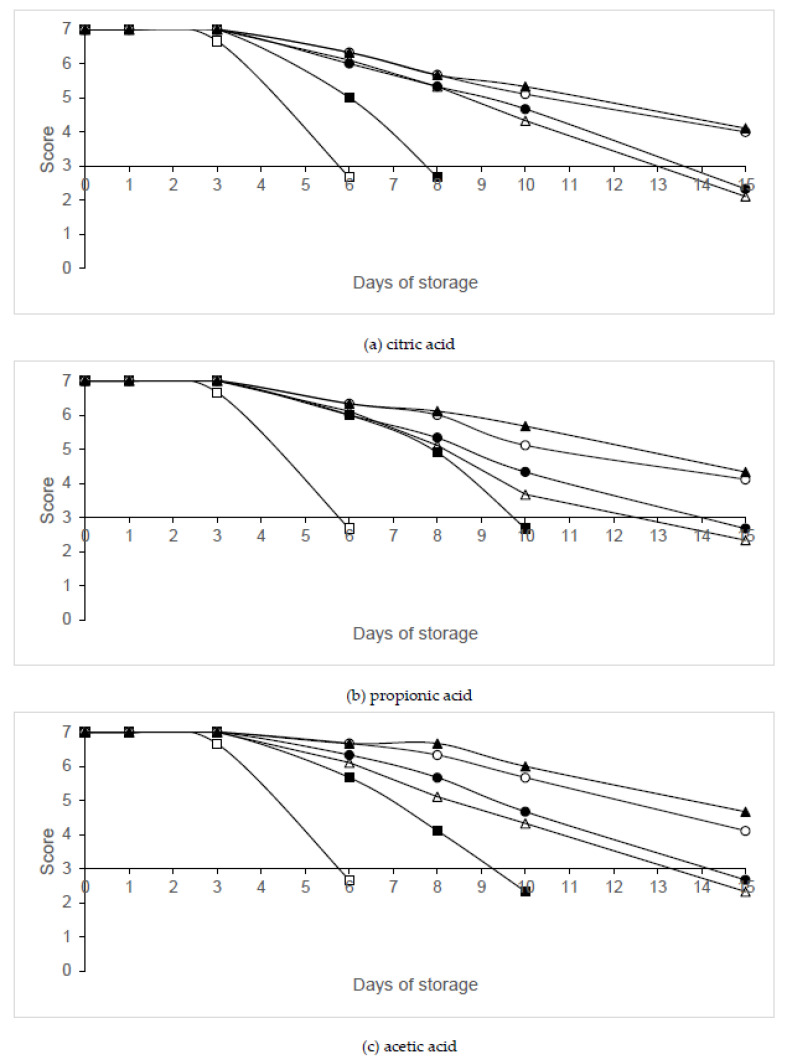
Evolution of sensorial scores in chicken legs treated with organic acids and packaged under modified atmospheres. Treatment conditions: Batch C, distilled water and packaging in air (□); 2% organic acid, packaging in air (▪); 2% organic acid, packaging in 80% N_2_/20% CO_2_ (∙); 2% organic acid, packaging in 60% N_2_/40% CO_2_ (◦); 2% organic acid, packaging in 40% N_2_/60% CO_2_ (▲); 2% organic acid, packaging under vacuum (Δ). (**a**) Citric acid; (**b**) propionic acid; (**c**) acetic acid. Data shown are the mean value of six determinations.

The sensorial acceptability of legs washed with organic acids and packaged under MAP is given in Figure 9. No significant (*p* > 0.05) effect on overall appearance were observed on days 0 and 1 between the different treatments. Legs that were packaged in air and not washed with organic acids were unacceptable on day 6, while those washed with organic acids were rejected on day 8 (citric acid) or 10 (acetic and propionic acid). The packaging in 60% N_2_/40% CO_2_ or 40% N_2_/60% CO_2_ in combination with citric, propionic or acetic acid resulted in a longer shelf life of chicken legs by at least 5 days compared to poultry legs washed with organic acids and packaged in air.

## 4. Discussion

Atmospheres with 60–80% N_2_ and 20–40% CO_2_ are usually used when packaging chicken [12]. In the current work, the effects of four atmospheres, namely 80% N_2_/20% CO_2_, 60% N_2_/40% CO_2_, 40% N_2_/60% CO_2_ and vacuum, were evaluated. The use of atmospheres containing CO_2_ or vacuum retarded the period to reach psychrotroph populations of 9 log cfu/g. Our data indicate that the atmosphere with the highest CO_2_ concentration (40% N_2_/60% CO_2_) was the most effective in decreasing the growth of psychrotrophs and extending the shelf life of chicken legs. Carbon dioxide has a more marked antimicrobial effect against psychrotrophic Gram-negative bacteria such as *Pseudomonas*, resulting in retarding the beginning of microbial spoilage [13]. Our data are in line with those presented by Arvanitoyannis and Stratakos [15], who reported that CO_2_ affects microorganisms by causing a decrease in the growth rate and an extension of the lag phase. Other researchers have also shown the reduction of psychrotrophs in poultry and meat storage under modified atmospheres [16,17].

The ability of *L. monocytogenes* to grow on chicken packaged in enriched CO_2_ atmospheres has also been pointed out by other authors [18,19]. In the current work, it was observed that the maximum growth rate of psychrotrophs in chicken legs packaged in MAP was lower than that of *L. monocytogenes*. Moreover, no lag phase for *L. monocytogenes* was detected in chicken samples packaged in modified atmospheres, while an extended lag phase was observed for psychrotrophs. Moreover, Marshall et al. [20] reported that *L. monocytogenes* can grow faster than spoilage bacteria in poultry meat packaged in MAP. Franco-Abuin et al. [19] found that an atmosphere containing 100% CO_2_ is more effective for the inhibition of growth of *L. monocytogenes* than other atmospheres with lower CO_2_ content. However, none of the gas mixtures was bactericidal. These findings suggest that strategies should be designed in order to control the growth of *L. monocytogenes* in poultry meat packaged in MAP.

Other authors have also observed a similar development of gas atmosphere in chicken filets packaged under MAP and kept at 4 °C [16,21]. Due to the high solubility of CO_2_ in tissues, a small reduction of CO_2_ was detected in all the packages over the first 2 days [22]. Our findings are in line with those of García de Fernando et al. [23], who reported a decrease in pH in meat packaged in atmospheres containing a high CO_2_ concentration (40–60% CO_2_).

Combinations of citric, acetic or propionic acid with packaging in modified atmospheres were effective in decreasing the growth of psychrotrophs, reducing their maximum growth rate and prolonging their lag phase in chicken meat. These treatments were more effective than treatments with these organic acids in nonpackaged chicken meat. In an earlier study, it was found that washing legs with 2% citric acid for 5 min decreased psychrotroph growth by between 0.58 and 1.1 log units compared with the control samples [8]. Cutter and Siragusa [24] also reported the efficacy of citric acid in reducing psychrotroph growth in beef. In the current study, the washing with 2% citric acid and packaging in 40% N_2_/60% CO_2_ decreased psychrotroph growth by between 0.99 and 3.12 log units compared to the control samples (nontreated and packaged in air), while the reductions in legs washed with citric acid and packaged in air were between 0.74 and 1.1 log units. In another study, it was found that a treatment with 2% propionic acid for 5 min decreased psychrotroph growth by between 1.27 and 2.13 log units compared with the control samples [9]. In the current study, the washing with 2% propionic acid and packaging in 40% N_2_/60% CO_2_ decreased psychrotroph growth by between 1.11 and 2.9 log units compared to the control samples, while the reductions in legs washed with propionic acid and packaged in air were between 1.1 and 1.29 log units. Jimenez et al. [13] also found that the immersion of chicken breast in acetic acid reduced psychrotroph growth. In an earlier study, it was found that washing with 2% acetic acid for 5 min decreased psychrotroph growth by between 0.49 and 2.52 log units compared with the control samples throughout storage [10]. In the current study, nontreated legs packaged in 40% N_2_/60% CO_2_ showed a psychrotroph growth reduction of between 1.16 and 2.04 log units compared with the control samples, while reductions of between 0.91 and 2.19 log units were found in legs treated with acetic acid and packaged in air. The treatment with 2% acetic acid and packaging in 40% N_2_/60% CO_2_ decreased psychrotroph growth by between 2.51 and 4.99 log units compared to the control samples, representing an additional 0.76 unit reduction when compared to the separate treatments. It seems that treatment with acetic acid and packaging in 40% N_2_/60% CO_2_ had a synergistic effect in reducing psychrotroph growth in chicken meat.

Other researchers have also pointed out that the combination of MAP with organic acids can increase the inhibitory effect against bacteria. According to Jimenez et al. [13], 1% acetic acid decontamination and packaging in 30% N_2_/70% CO_2_ can reduce the growth of *Pseudomonas* and prolong the lag phase of lactic acid bacteria in chicken breasts. Sawaya et al. [25] pointed out that a pretreatment of poultry with lactic acid and packaging in 70% CO_2_ reduced psychrotroph growth and prolonged the shelf life of poultry. Djenane et al. [26] studied the effect of different modified atmospheres (40% CO_2_/60% O_2_ and 10% N_2_/20% CO_2_/70% O_2_) in combination with antioxidants (0.05% ascorbic acid and 0.1% rosemary) and lactic acid treatment on beef steaks. These authors observed that *Pseudomonas* spp. was inhibited by the treatment with lactic acid and the higher concentrations of CO_2_. The combination of organic acid treatments and CO_2_ packaging seems to be more effective against spoilage bacteria than the combination with vacuum packaging [27]. Sawaya et al. [25] showed that a combination of sorbate treatment and vacuum packaging decreased microbial growth and prolonged the shelf life of chicken carcasses. Other decontaminating agents in combination with MAP can have an enhanced inhibitory effect against bacteria in chicken meat [28,29].

The reduction of pH values of samples washed with organic acids has also been reported by other authors [28]. Sensory quality was not negatively affected by 2% citric, propionic or acetic acid washing or vacuum packaging [8,9,10,13,30,31].

The combination of modified atmosphere packaging (MAP) and organic acid treatment of chicken meat seems to be more effective in reducing *L. monocytogenes* than treatment of nonpackaged chicken with organic acids. In an earlier study, it was found that legs washed with 2% citric acid for 5 min showed a significant growth reduction in *L*. *monocytogene*s (between 0.22 and 1.12 log units compared with the control samples) [8]. Moreover, Menconi et al. [32] showed the efficacy of citric acid against *L. monocytogenes* in chicken skin. In the current study, the washing with 2% citric acid and packaging in 40% N_2_/60% CO_2_ reduced *L*. *monocytogene*s growth by between 1.13 and 2.15 log units compared to the control samples (nontreated and packaged in air) while no reduction of *L. monocytogenes* growth was found in legs packaged in 40% N_2_/60% CO_2_, and reductions of between 0.67 and 1.06 log units were found in legs treated with citric acid and packaged in air (1.09 units more than the separate treatments). In an earlier study, it was found that samples washed with 2% propionic acid for 5 min showed a significant decrease in *L*. *monocytogene*s growth, with reductions between of 1.89 and 2.72 log units compared with the control samples [9]. In the current study, the washing with 2% propionic acid and packaging in 40% N_2_/60% CO_2_ reduced *L*. *monocytogene*s growth by between 2.14 and 3.3 log units compared to the control samples (nontreated and packaged in air), while no reduction of *L. monocytogenes* growth was observed in legs packaged in 40% N_2_/60% CO_2_, and reductions of between 2.14 and 2.81 log units were found in legs washed with propionic acid and packaged in air (0.49 units more than the separate treatments). In an earlier study, it was found that legs treated with 2% acetic acid for 5 min showed a significant decrease in *L*. *monocytogene*s growth, with reductions of between 0.65 and 1.66 log units compared with the control samples [10]. Dorsa et al. [33] also observed that the treatment of beef with 1.5–3% acetic acid reduced *Listeria* growth. In the current study, the treatment with 2% acetic acid and packaging in 40% N_2_/60% CO_2_ decreased *L*. *monocytogene*s growth by between 0.88 and 2.09 log units compared to the control samples (nontreated and packaged in air), while no reduction in *L. monocytogenes* growth was observed in legs packaged in 40% N_2_/60% CO_2_, and growth reductions of between 0.88 and 1.87 log units were found in legs treated with acetic acid and packaged in air (0.22 units more than the separate treatments). It seems that treatment with citric, propionic and acetic acids and packaging in 40% N_2_/60% CO_2_ had a synergistic effect in reducing *L*. *monocytogene*s growth in chicken meat.

After treatment with organic acids (day 0), the most effective treatment against *L*. *monocytogene*s was the propionic acid. Greater growth reductions of *L*. *monocytogene*s counts were obtained in legs washed with propionic acid than in those washed with acetic or citric acid. Moreover, Cunningham et al. [34] observed that propionic acid was more effective in reducing *L*. *monocytogene*s growth than acetic acid in broth media. However, the lowest growth rates for *L*. *monocytogene*s were found in legs washed with 2% acetic acid and packaged in atmospheres containing CO_2_. These findings could be explained by the residual activity displayed by acetic acid preventing microbial growth [35]. The efficacy of organic acids against *L*. *monocytogene*s has been pointed out by other authors [36,37]. Lues and Theron [36] studied the minimum inhibitory concentration of different organic acids against *L*. *monocytogene*s in culture media. According to these authors, at a pH value of 6.5, lower minimum inhibitory concentration (MIC) levels (high susceptibility) were found for acetic, citric and propionic acid than for lactic or malic acid. We observed that propionic and acetic acids were more effective than citric acid in controlling *L. monocytogenes* in chicken meat. According to Ahamad and Marth [37], acetic acid is the more effective against *L. monocytogenes* in culture media than lactic and citric acids. The activity of organic acids against *L. monocytogenes* could be related to their degree of undissociation. Citric and lactic acids have higher dissociation constants, being less detrimental to *L. monocytogenes* than acetic and propionic acids are. Other authors have also pointed out that propionic acid was more effective than citric acid against *Salmonella* in culture media [38]. According to these authors, the maximum inhibition against *Salmonella* was exerted by propionic acid, followed by lactic, acetic and citric acids [39].

Our results suggest that in high CO_2_ environments, organic acids might act synergistically to reduce the growth of *L. monocytogenes*. The efficacy of organic acids and MAP in *L. monocytogenes* control in meat and poultry has been also pointed out by other authors [7,39,40,41]. The effect of lactic acid and MAP on *L. monocytog*enes in chicken was investigated by Zeitoun and Debevere [7]. These authors pointed out that the best results against *L. monocytogenes* were obtained by the combination of MAP and 10% lactic acid. Spraying or dipping of cured meat products in an organic acid solution prior to packaging has been found to reduce *L. monocytogenes* growth [39]. Glass et al. [40] reported that the use of propionic acid in turkey products stored under vacuum inhibited *L. monocytogenes* growth. Additionally, Murphy et al. [41] pointed out the efficacy of an organic solution containing 2% acetic acid in controlling *L. monocytogenes* in meat products packaged under vacuum.

## 5. Conclusions

Chicken legs packaged in 40% N_2_/60% CO_2_ had a prolonged shelf life, but these conditions were not able to decrease *L. monocytogenes* populations; consequently, strategies should be designed in order to control this bacterial pathogen. The treatment with acetic acid was effective in decreasing the growth of psychrotrophs, decreasing their maximum growth rate and extending their lag phase in chicken legs packaged in atmospheres containing 60% N_2_/40% CO_2_ or 40% N_2_/60% CO_2_. Washing chicken legs with 2% acetic acid can decrease *L. monocytogenes* populations in chicken packaged in MAP. The treatment with acetic acid was effective in reducing *L. monocytogenes* growth, decreasing their maximum growth rate and extending their lag phase in chicken legs packaged in atmospheres containing 60% N_2_/40% CO_2_ or 40% N_2_/60% CO_2_. Chicken meat can be contaminated with *L. monocytogenes* during processing and packaging, and thus it is necessary to reduce populations of this bacterial pathogen and control its growth during refrigeration storage. The treatment with acetic acid could help to control this pathogen.

## Figures and Tables

**Table 1:** Washing treatment and atmosphere composition of different chicken leg batches.

Batch	Washing Treatment	Atmosphere
C	distilled water	Air
CA	2% citric acid	Air
CAMA20	2% citric acid	80% N_2_/20% CO_2_
CAMA40	2% citric acid	60% N_2_/40% CO_2_
CAMA60	2% citric acid	40% N_2_/60% CO_2_
CAMAV	2% citric acid	Vacuum
PA	2% propionic acid	Air
PAMA20	2% propionic acid	80% N_2_/20% CO_2_
PAMA40	2% propionic acid	60% N_2_/40% CO_2_
PAMA60	2% propionic acid	40% N_2_/60% CO_2_
PAMAV	2% propionic acid	vacuum
AA	2% acetic acid	air
AAMA20	2% acetic acid	80% N_2_/20% CO_2_
AAMA40	2% acetic acid	60% N_2_/40% CO_2_
AAMA60	2% acetic acid	40% N_2_/60% CO_2_
AAMAV0	2% acetic acid	vacuum

**Table 2:** Growth parameters of psychrotrophs and *L. monocytogenes* in packaged chicken.

Batch	Psychrotrophs			*L. monocytogenes*		
	λ	µ_max_	R^2^	λ	µ_max_	R^2^
A0	-	0.685 ± 0.174 ^a^	0.953	-	0.745 ±0.079 ^a^	0.988
A20	0.249 ± 0.693 ^a^	0.528 ± 0.053 ^a^	0.992	-	0.588 ±0.050 ^bc^	0.987
A40	0.779 ± 0.557 ^a^	0.468 ±0.040 ^b^	0.994	-	0.578 ± 0.035 ^bc^	0.994
A60	1.654 ±0.425 ^b^	0.452 ±0.034 ^b^	0.995	-	0.543 ± 0.028 ^c^	0.995
AV	0.717 ± 0.77 ^a^	0.547 ±0.063 ^a^	0.992	-	0.618 ± 0.035 ^b^	0.994

Mean ± standard deviation, Means in the same column with no superscript letters in common are significantly different (*p* < 0.05). **λ**, lag phase (day); µ_max_, maximum growth rate (log cfu/g/day); R^2^, coefficient of determination. Packaging conditions: A0, air; A20, 80% N_2_/20% CO_2_; A40, 60% N_2_/40% CO_2_; A60, 40% N_2_/60% CO_2_; AV, vacuum.

**Table 3:** Growth parameters of psychrotrophs and *L. monocytogenes* in chicken immersed in different organic acids (citric, propionic and acetic acids) and packaged under modified atmospheres.

Organic Acid	Batch	Psychrotrophs			*L. monocytogenes*		
		λ	µ_max_	R^2^	λ	µ_max_	R^2^
	C	-	0.660 ± 0.074^a^	0.991	-	0.787 ± 0.036 ^a^	0.997
Citric acid	CA	-	0.684 ± 0.057^a^	0.989	-	0.795 ± 0.132 ^a^	0.975
	CAMA20	1.194 ± 1.168 ^a^	0.558 ± 0.097 ^ab^	0.972	-	0.497 ± 0.055 ^b^	0.968
	CAMA40	1.336 ± 0.521 ^a^	0.448 ± 0.030 ^bc^	0.995	0.662 ± 0.771 ^a^	0.608 ± 0.096 ^b^	0.969
	CAMA60	1.825 ± 1.192 ^a^	0.413 ± 0.067^bc^	0.972	0.708 ± 0.595 ^a^	0.512 ± 0.043 ^bc^	0.985
	CAMAV	0.715 ± 1.669 ^a^	0.521 ± 0.108 ^ab^	0.953	-	0.536 ± 0.058 ^bc^	0.973
Propionic acid	PA	-	0.606 ± 0.017^a^	0.997	0.257 ± 0.380 ^a^	0.590 ± 0.033 ^b^	0.997
	PAMA20	0.553 ± 0.710 ^a^	0.569 ± 0.049^ab^	0.992	0.492 ± 0.261 ^a^	0.589 ± 0.019 ^b^	0.999
	PAMA40	0.868 ± 0.718 ^a^	0.520 ± 0.047^b^	0.987	0.634 ± 0.289 ^a^	0.507 ± 0.021 ^bc^	0.998
	PAMA60	1.135 ± 1.754 ^a^	0.321 ± 0.029^d^	0.951	0.765 ± 0.567 ^a^	0.508 ± 0.047 ^bc^	0.993
	PAMAV	0.773 ± 0.703 ^a^	0.427 ± 0.093^cb^	0.989	0.589 ± 0.255 ^a^	0.597 ± 0.024 ^b^	0.999
Acetic acid	AA	1.408 ± 1.307 ^a^	0.566 ± 0.088^ab^	0.960	-	0.516 ± 0.074 ^bc^	0.953
	AAMA20	2.303 ± 0.707 ^a^	0.386 ± 0.025^c^	0.990	0.602 ± 1.878 ^a^	0.348 ± 0.069 ^d^	0.895
	AAMA40	4.873 ± 0.927 ^b^	0.420 ± 0.047^c^	0.968	0.750 ± 1.772 ^a^	0.338 ± 0.064 ^d^	0.933
	AA60 d17	5.224 ± 1.349 ^b^	0.409 ± 0.052^c^	0.954	0.780 ± 1.963 ^a^	0.309 ± 0.065 ^d^	0.886
	AAMAV	2.096 ± 0.433 ^a^	0.396 ± 0.015^c^	0.996	0.748 ± 1.290 ^a^	0.488 ± 0.067 ^c^	0.939

Mean ± standard deviation. Means in the same column with no superscript letters in common are significantly different (*p* < 0.05). **λ,** lag phase (day); µ_max_, maximum growth rate (log cfu/g/day); R^2^, coefficient of determination. Treatment conditions: Batch C, distilled water and packaging in air; Batch CA, 2% citric acid and packaging in air; Batch CAMA20, 2% citric acid and packaging in 80% N_2_/20% CO_2_; Batch CAMA40, 2% citric acid and packaging in 60% N_2_/40% CO_2_; Batch CAMA60, 2% citric acid and packaging in 40% N_2_/60% CO_2_; Batch CAMAV, 2% citric acid and packaging under vacuum.
